# Inflammation and micronutrient biomarkers predict clinical HIV treatment failure and incident active TB in HIV-infected adults: a case-control study

**DOI:** 10.1186/s12916-018-1150-3

**Published:** 2018-09-24

**Authors:** Rupak Shivakoti, Nikhil Gupte, Srikanth Tripathy, Selvamuthu Poongulali, Cecilia Kanyama, Sima Berendes, Sandra W. Cardoso, Breno R. Santos, Alberto La Rosa, Noluthando Mwelase, Sandy Pillay, Wadzanai Samaneka, Cynthia Riviere, Patcharaphan Sugandhavesa, Robert C. Bollinger, Ashwin Balagopal, Richard D. Semba, Parul Christian, Thomas B. Campbell, Amita Gupta, for the NWCS 319 and PEARLS Study Team

**Affiliations:** 1 Department of Medicine, Johns Hopkins University School of Medicine, Baltimore, MD USA; 2 National AIDS Research Institute, Pune, India; 3 YR Gaitonde Center for AIDS Research and Education, Chennai, India; 4 UNC Lilongwe, Lilongwe, Malawi; 5 Malawi College of Medicine-Johns Hopkins University Research Project, Blantyre, Malawi; 6 STD/AIDS Clinical Research Laboratory, Instituto de Pesquisa Clinica Evandro Chagas, Fundacao Oswaldo Cruz, Rio de Janeiro, Brazil; 7 Hospital Nossa Senhora de Conceiçã, Porto Alegre, Brazil; 8 Asociacion Civil Impacta Salud y Educacion, Lima, Peru; 9 Department of Medicine, University of Witwatersrand, Johannesburg, South Africa; 10 Durban International Clinical Research Site, Durban University of Technology, Durban, South Africa; 11 University of Zimbabwe Clinical Research Centre, Harare, Zimbabwe; 12 Les Centres GHESKIO, Port-Au-Prince, Haiti; 13 Research Institute for Health Sciences, Chiang Mai, Thailand; 14 Department of Ophthalmology, Johns Hopkins University School of Medicine, Baltimore, MD USA; 15 Department of International Health, Johns Hopkins Bloomberg School of Public Health, Baltimore, MD USA; 16 Department of Medicine, Division of Infectious Diseases, University of Colorado School of Medicine, Aurora, CO USA; 17 Present Address: Department of Epidemiology, Columbia University Mailman School of Public Health, 722 West 168th St, Room 705, New York, NY 10032 USA; 18 Present Address: National Institute of Research in Tuberculosis, Chennai, India; 19 Present Address: Liverpool School of Tropical Medicine, Liverpool, UK; 20 Present Address: Bill and Melinda Gates Foundation, Seattle, USA

**Keywords:** HIV, Inflammation, Antiretroviral therapy, Tuberculosis, IL-18, Exploratory factor analysis

## Abstract

**Background:**

Various individual biomarkers of inflammation and micronutrient status, often correlated with each other, are associated with adverse treatment outcomes in human immunodeficiency virus (HIV)-infected adults. The objective of this study was to conduct exploratory factor analysis (EFA) on multiple inflammation and micronutrient biomarkers to identify biomarker groupings (factors) and determine their association with HIV clinical treatment failure (CTF) and incident active tuberculosis (TB).

**Methods:**

Within a multicountry randomized trial of antiretroviral therapy (ART) efficacy (PEARLS) among HIV-infected adults, we nested a case-control study (*n* = 290; 124 cases, 166 controls) to identify underlying factors, based on EFA of 23 baseline (pre-ART) biomarkers of inflammation and micronutrient status. The EFA biomarker groupings results were used in Cox proportional hazards models to study the association with CTF (primary analysis where cases were incident World Health Organization stage 3, 4 or death by 96 weeks of ART) or incident active TB (secondary analysis).

**Results:**

In the primary analysis, based on eigenvalues> 1 in the EFA, three factors were extracted: (1) carotenoids), (2) other nutrients, and (3) inflammation. In multivariable-adjusted models, there was an increased hazard of CTF (adjusted hazard ratio (aHR) 1.47, 95% confidence interval (CI)1.17–1.84) per unit increase of inflammation factor score. In the secondary incident active TB case-control analysis, higher scores of the high carotenoids and low interleukin-18 factor was protective against incident active TB (aHR 0.48, 95% CI 0.26–0.87).

**Conclusion:**

Factors identified through EFA were associated with adverse outcomes in HIV-infected individuals. Strategies focused on reducing adverse HIV outcomes through therapeutic interventions that target the underlying factor (e.g., inflammation) rather than focusing on an individual observed biomarker might be more effective and warrant further investigation.

**Electronic supplementary material:**

The online version of this article (10.1186/s12916-018-1150-3) contains supplementary material, which is available to authorized users.

## Background

Single biomarkers have been assessed in antiretroviral therapy (ART)-naïve HIV-infected adults in multiple studies, and results show that specific biomarkers of inflammation or micronutrient concentrations are associated with adverse outcomes [1–4]. Various markers of inflammation, including C-reactive protein (CRP), soluble CD14 (sCD14), and various cytokines (e.g., interleukin-6 and 18 (IL-6 and IL-18)), are associated with increased mortality and morbidity [1–6]. Morbidity includes increased clinical treatment failure (CTF), risk of incident active TB, and even longer-term outcomes such as cardiovascular disease. Similarly, studies have shown that levels of various micronutrients such as vitamin D, selenium, and iron can also affect various HIV outcomes [7–11].

However, many of the inflammation markers are correlated with each other, such as in cases where they might be activated by the same stimuli or signaling pathway [12]. The nutritional biomarkers might also correlate with each other, for example, if different micronutrients are part of the same or similar foods [13]. Furthermore, there is evidence that inflammation and micronutrient status can directly affect each other. For example, studies show that circulating levels of selenium and zinc are reduced during the acute phase response [14, 15]. As a result, studies on the association of these biomarkers with outcomes might benefit from considering the relationship between these biomarkers.

Data reduction methods, such as exploratory factor analysis (EFA), have been useful in dealing with multicollinearity [16]. EFA is a statistical method for data reduction, in which numerous observed variables (e.g., circulating biomarkers) that co-vary with each other are assumed to reflect a smaller number of underlying unobserved variables (“factors”) [17]. An important point to note for these analyses is that each individual will receive a score for each factor (i.e., an individual whose pattern poorly matches the factor gets a lower score), and the association of a specific factor with an outcome is compared between individuals with high and low scores of that factor, rather than between different factors.

Various studies have shown that factor analysis can identify biologically meaningful “biomarker superfamilies” which can be utilized to stratify individuals in high- and low-risk subgroups [12, 18–20] based on their factor score and potentially identify therapeutics that target the underlying factor rather than the individual observed variables. An example of the public health potential for EFA is evident in the field of nutrition and obesity where dietary patterns (e.g., high fat/low fiber pattern) are identified based on EFA and are part of the intervention (e.g., to consume less of that pattern) [21, 22].

The goal of this study was to conduct EFA on multiple observed biomarkers of inflammation and micronutrient status in HIV-infected adults initiating ART, in order to identify biomarker groupings that are hypothesized to represent underlying biological processes (factors) and to determine whether these factors were associated with adverse HIV outcomes including CTF (CTF as primary outcome) and incident active tuberculosis (TB) (TB as secondary outcome). To address this research question, we conducted nested case-control studies within a multicountry randomized clinical trial of ART efficacy (PEARLS) [23].

## Methods

### Study design and population

PEARLS was conducted from 2005 to 2010 (NCT00084136) [23] in 1571 ART-naïve HIV-infected adults from diverse settings to compare the efficacy of three different ART regimens: (1) efavirenz plus twice-daily lamivudine-zidovudine; (2) atazanavir plus didanosine EC and emtricitabine, all given once daily; or (3) efavirenz plus emtricitabine-tenofovir DF once daily. The primary efficacy outcome was treatment failure. PEARLS trial participants who met the inclusion criteria (including age greater than 18 years old and CD4+ T cell count less than 300 cells/mm^3^) were recruited from nine different countries: Brazil (*n* = 231), Haiti (*n* = 100), India (*n* = 255), Malawi (*n* = 221), Peru (*n* = 134), South Africa (*n* = 210), Thailand (*n* = 100), the USA (*n* = 210), and Zimbabwe (*n* = 110). Pregnant women and individuals with an acute illness or severe anemia were excluded from the study. Detailed inclusion and exclusion criteria for PEARLS are described elsewhere [23].

For this study, we nested a case-control analysis (*n* = 290; 124 cases, 166 controls) within PEARLS to assess the association of baseline (pre-ART initiation) biomarkers of inflammation and micronutrients with CTF. CTF was defined as an incident World Health Organization (WHO) stage 3 or 4 event (including incident active TB) or death within 96 weeks post-ART initiation [24]. While all the cases from the parent study with available biomarker values were selected, controls (who did not develop clinical failure by 96 weeks) were selected based on random subsampling of the parent cohort stratified by country (the same approach used for secondary case controls).

Three other secondary nested case-control analyses were performed: severe outcome case control (*N* = 254; 81 cases and 173 controls), virologic failure case control (*N* = 260; 90 cases and 170 controls), and incident active TB case control (*N* = 220; 47 cases and 173 controls) analyses. The outcomes analyzed for these case controls were (1) severe outcomes defined as death, serious bacterial infections or sepsis, and opportunistic infections (including TB), (2) virologic failure defined as HIV-1 RNA levels ≥ 1000 copies/mL for two successive visits at ≥ 16 weeks after ART initiation, and (3) incident active TB defined as pulmonary or extrapulmonary TB that developed during the follow-up period (96 weeks post-ART initiation). We use the term “incident active TB” to define anyone who presented with signs or symptoms of TB disease and resulted in having confirmed or probable TB disease after entry into the study; the term does not distinguish between recently acquired TB through transmission, reactivation of TB, and subclinical/unmasked TB. Using standardized AIDS Clinical Trials Group (ACTG) definitions, incident active TB was defined as one of the following: confirmed pulmonary TB, probable pulmonary TB, confirmed extrapulmonary TB, probable extrapulmonary TB, and TB immune reconstitution inflammatory syndrome (TB-IRIS). While the diagnostic information is described in detail elsewhere [6], cases were considered confirmed if TB was isolated by culture, and they were considered probable based on signs and symptoms, acid-fast bacilli stain, x-rays, and TB treatment initiation. The definitions were standardized across the various sites, and the data for each diagnosis were reviewed by five physicians in the study team.

### Data collection and laboratory analysis

Clinical history, including outcome assessment, was collected at baseline and at 2, 4, and 8 weeks post-ART initiation. After 8 weeks, clinical history was collected every 4 weeks through 24 weeks and every 8 weeks after that through 96 weeks. Plasma and serum samples were collected at baseline and other relevant time points and stored at − 80 °C.

The exposure variables (23 markers of inflammation and micronutrient status) were measured from plasma and serum samples collected at baseline (pre-ART initiation). Inflammation markers assessed in this study were interferon-γ (IFN-γ), IL-6, IFN-γ inducible protein (IP)-10, IL-18, tumor necrosis factor-α (TNF-α), CRP, sCD14, and EndoCAb immunoglobulin M (IgM). Luminex multiplex enzyme-linked immunosorbent assays (ELISAs) (R&D Systems, Minneapolis, MN, USA) were used to measure plasma levels of IFN-γ, IL-6, and TNF-α, while IP-10 was measured by MSD multiplex ELISAs (Meso Scale Discovery, Rockville, MD, USA). Single-plex ELISAs were used to measure plasma CRP, sCD14 (both R&D Systems), IL-18 (eBiosciences), and EndoCAb IgM (Cell Sciences, Canton, MA, USA). Further details on the inflammation markers are described elsewhere [6, 25].

The micronutrient markers assessed in this study were markers of vitamin A (retinol), vitamin B_6_, vitamin B_12_, vitamin D, vitamin E (α-tocopherol and γ-tocopherol), iron (ferritin and soluble transferrin receptor), selenium, and various carotenoids (α-carotene, β-carotene, β-cryptoxanthin, lutein, lycopene, and zeaxanthin). Details of assessment are described elsewhere [26]. Briefly, serum ferritin (ALPCO, Salem, NH, USA) and soluble transferrin receptor (R&D Systems) were measured using an ELISA, while radioimmunoassay (DiaSorin, Saluggia, Italy) was used to measure total (D_2_ and D_3_) serum 25-hydoxyvitamin. High-performance liquid chromatography (HPLC) was used to measure serum vitamin B_6_, retinol (vitamin A), and the carotenoids, as previously described [26]. Abbott AxSYM (Abbott Laboratories, Lake Bluff, IL, USA), an automated immunochemical analyzer, was used to measure serum vitamin B_12_, while serum selenium was assessed with a Perkins-Elmer AAnalyst 600 graphite furnace atomic absorption spectrometer.

Potential confounders measured at baseline and included in multivariable models were body mass index (BMI), CD4+ T cell count, plasma HIV RNA, hemoglobin, and albumin. Plasma HIV-1 RNA was measured using the Roche Amplicor Monitor Assay (v1.5; Roche Molecular Systems, Branchburg, NJ, USA). Serum hemoglobin, albumin, and CD4+ T cell count were measured in the individual site laboratories that met the quality assurance standards of the NIH ACTG Network laboratory [23].

### Statistical analyses

Twenty-three immune and micronutrient biomarkers were used to perform EFA, and the principal factor method was used, which is the default factor analysis method utilized by STATA software. In the principal factor method, the factor loadings are calculated using squared multiple correlations. While we also considered the proportion of variance explained, the final numbers of factors were extracted based on scree plots and eigenvalues (number of observed variables which the factor represents) greater than 1, as commonly done in EFA studies [27]. For simpler interpretation, factors were treated as orthogonal (where it is assumed that the factors themselves are not correlated to each other) and were rotated through the varimax method, which improves interpretability of orthogonal factors by rotating the axes so that each observed variable will load strongly on one of the factors [12]. Factor loadings above 0.3 were considered significant [28]; loadings are regression coefficients that describe the relationship between an unmeasured variable (i.e., an underlying factor which is not directly measured) and an observed/measured biomarker (a similar idea to correlation but not the same). The common characteristics among the high loading biomarkers for each factor were used for interpreting and naming the factors.

Based on the EFA, factor scores were generated for each participant, with higher scores indicating a higher standing in the scale (i.e., a better match to that factor). The factor scores of the individuals were used in univariable and multivariable Cox proportional hazard models to determine the association of each factor with outcome (CTF, severe outcomes, or TB). Sex, age, country, BMI, baseline TB status, CD4 count, viral load, treatment arm (ART regimen), anemia, and hypoalbuminemia were adjusted for in multivariable models. Baseline TB status is a binary variable referring to prior (i.e., the patient had TB disease and TB treatment prior to study initiation) or prevalent TB (i.e., under TB treatment at baseline). Race was not used in multivariable models due to co-linearity with country. STATA version 13 was used for data analysis.

Note that although the source population for the severe outcomes and TB case controls was the same PEARLS study as for the CTF case control, the specific study population for each case control is different (due to the different case definitions). As a result, although factors are extracted independent of the outcome, the different case controls themselves could have different factor profiles since each case control has a different sample population.

## Results

### Study population characteristics

The baseline characteristics of the cases and controls in the CTF case-control population differed significantly by the following baseline characteristics: country (*p* = 0.001), BMI (*p* = 0.001), prior TB diagnosis (*p* = 0.01), CD4 T cell count, hypoalbuminemia, and anemia (*p* < 0.001 for all) (Table 1).

**Table 1:** Characteristics of population by CTF cases and control status

Characteristic	All *n* = 290	CTF *n* = 124 (43%)	Controls *n* = 166 (57%)	*p* value^a^
Gender				
Male	160 (55)	71 (44)	89 (56)	0.55
Female	130 (45)	53 (41)	77 (59)	
Age (years)	35.0 (29.0–42.0)	35.5 (29.5–42.0)	35.0 (29.0–42.0)	0.81
Country				
Brazil	44 (15)	16 (36)	28 (64)	0.001
Haiti	34 (12)	11 (32)	23 (68)	
India	23 (8)	18 (78)	5 (22)	
Malawi	38 (13)	22 (58)	16 (42)	
Peru	19 (7)	4 (21)	15 (79)	
South Africa	38 (13)	20 (53)	18 (47)	
Thailand	23 (8)	6 (26)	17 (74)	
USA	44 (15)	17 (39)	27 (61)	
Zimbabwe	27 (9)	10 (37)	17 (63)	
Body mass index (kg/m^2^)				
< 18.5	29 (10)	18 (62)	11 (38)	0.001
18–25	192 (66)	89 (46)	103 (54)	
≥ 25	69 (24)	17 (25)	52 (75)	
Prior TB diagnosis				
Yes	59 (20)	34 (58)	25 (42)	0.01
No	231 (80)	90 (39)	141 (61)	
Treatment arm				
A	100 (35)	58 (56)	45 (44)	0.72
B	108 (37)	54 (42)	39 (58)	
C	82 (28)	67 (29)	27 (71)	
CD4 count (cells/mm^3^)				
< 100	103 (36)	50 (69)	22 (31)	< 0.001
100–200	93 (32)	45 (49)	46 (51)	
> 200	94 (34)	43 (41)	63 (59)	
Log viral load (copies/mL)				
< 4	21 (7)	6 (29)	15 (71)	0.18
4–5	98 (34)	38 (39)	60 (61)	
> 5	171 (59)	80 (47)	91 (53)	
Hypoalbuminemia				
Yes (≤ 3.5 g/dL)	77 (19)	51 (41)	26 (16)	< 0.001
No (> 3.5 g/dL)	215 (81)	73 (59)	140 (84)	
Anemia				
Yes	168 (58)	87 (54)	81 (46)	< 0.001
No	121 (42)	36 (50)	85 (50)	

Data are presented as number (%) of the CTF case control. Anemia is defined based on hemoglobin cutoffs for males (< 13.0 g/dL) and non-pregnant females (< 12.0 g/dL). ^a^Fisher’s exact test was used to calculate the *p* values for categorical variables, and the rank sum test for continuous variables

### EFA and association with clinical treatment failure

From the CTF case-control EFA analysis, three underlying factors (Additional file 1: Table S1) were extracted based on correlations among the observed biomarkers (eigenvalues > 1). In an EFA, the names of the factors are given by the researchers after carefully examining what is common between the observed variables/biomarkers that have high loadings (correlation equivalent) on each factor.

Factor 1 (carotenoids) had high factor loadings (> 0.30) of carotenoids including α-carotene, β-carotene, β-cryptoxanthin, lutein, and zeaxanthin. Factor 2 (other nutrients) had high loadings of selenium, vitamin B_6_, vitamin E (α-tocopherol), lycopene, α-carotene, and β-cryptoxanthin (the last three are carotenoids as well). Factor 3 (inflammation) had high loadings of C-reactive protein (CRP), soluble CD14 (sCD14), interleukin 18 (IL-18), and ferritin (an indicator of iron stores but also an acute phase protein) (Additional file 1: Table S1). As is common in factor analysis, some biomarkers (e.g., vitamin D) did not have high loadings in any of the three extracted factors.

Higher scores of the carotenoids and other nutrients factors were associated with reduced hazards of CTF in univariable models, but not in multivariable models that adjusted for sex, age, country, BMI, baseline TB status, CD4 count, viral load, treatment arm, anemia, and hypoalbuminemia (Table 2). In contrast, higher scores of inflammation factor were associated with increased hazards of CTF in both univariable (hazard ratio (HR) 1.53; 95% confidence interval (CI) 1.32–1.77) and multivariable (adjusted HR (aHR) 1.47; 95% CI 1.17–1.84) analyses (Table 2).

**Table 2:** Association of each factor with clinical treatment failure

	Univariable analysis HR (95% CI)	Multivariable analysis HR (95% CI)
Factor 1 (Carotenoids)	0.71 (0.56–0.90)	0.77 (0.57–1.05)
Factor 2 (Other nutrients)	0.79 (0.63–0.87)	0.83 (0.57–1.32)
Factor 3 (Inflammation)	1.53 (1.32–1.77)	1.47 (1.17–1.84)

The association of each factor with CTF was determined in univariable and multivariable Cox regression models. Sex, age, country, treatment arm, body mass index (BMI), baseline TB status, CD4 count, viral load, anemia, and hypoalbuminemia were adjusted for in the multivariable models. *N* = 290 (124 cases, 166 controls)

As CTF is a composite diagnosis that includes multiple outcomes with a range in severity, we also assessed whether similar patterns were observed when we limited our analysis to more severe outcomes. In the severe outcome case-control population, cases and controls differed significantly by the following baseline characteristics: country (*p* = 0.01), BMI (*p* = 0.003), prior TB (*p* = 0.02), CD4 count, hypoalbuminemia, and anemia (all *p* < 0.001) (data not shown). For this severe outcome analysis, three factors were extracted based on the EFA (eigenvalues > 1). The factors extracted in this analysis had a remarkably similar profile to our prior analysis where CTF was the outcome (Additional file 2: Table S2). Factor 1 (carotenoids) and factor 2 (other nutrients) had high loadings of the same markers, while factor 3 (inflammation) had an additional marker with high loading (IP-10) (Additional file 2: Table S2). Similar to our analysis with CTF, higher scores of the carotenoids and other nutrients factor were associated with reduced hazards of severe outcomes in univariable but not multivariable models (Table 3). In contrast, higher scores of the inflammation factor had increased hazards of severe outcomes only in multivariable models (aHR 1.60; 95% CI 1.24–2.06) (Table 3).

**Table 3:** Association of each factor with severe outcomes

	Univariable analysis HR (95% CI)	Multivariable analysis HR (95% CI)
Factor 1 (Carotenoids)	0.71 (0.52–0.95)	0.86 (0.58–1.26)
Factor 2 (Other nutrients)	0.75 (0.57–0.98)	0.78 (0.49–1.26)
Factor 3 (Inflammation)	0.97 (0.61–1.53)	1.60 (1.24–2.06)

The association of each factor with severe outcomes was determined in univariable and multivariable Cox regression models. Sex, age, country, treatment arm, body mass index (BMI), baseline TB status, CD4 count, viral load, anemia, and hypoalbuminemia were adjusted for in the multivariable models. Severe outcomes were defined as death, serious bacterial infections/sepsis, and opportunistic infections. *N* = 254 (81 cases, 173 controls)

In addition to severe outcomes, we also conducted a virologic failure case-control analysis. The factor profiles were very similar to those of the CTF and severe outcomes analysis, where three factors were extracted and had the same high loading markers (Additional file 3: Table S3). As with the CTF analysis, higher scores of the inflammation factor (but not the other two factors) were associated with increased hazards of virologic failure in both univariable (HR 1.32; 95% CI 1.11–1.56) and multivariable models (aHR 1.36; 95% CI 1.05–1.75) (Additional file 4: Table S4).

### EFA and association with incident active TB

In the incident active TB case-control population, cases and controls differed significantly by the following baseline characteristics: BMI (*p* = 0.001), prior TB (*p* = 0.02), CD4 count (*p* = 0.02), country, hypoalbuminemia, and anemia (all *p* < 0.001) (data not shown). The EFA analysis of baseline biomarkers in the incident active TB case-control analysis also yielded three factors (eigenvalues > 1). However, the profiles of the factors and their association with incident active TB had some important differences from the CTF analyses (Additional file 5: Table S5). In this analysis, factor 1 (high carotenoids and low IL-18) had high loadings of α-carotene, β-carotene, β-cryptoxanthin, lutein, and zeaxanthin, as well as high negative loadings of IL-18, ferritin, and γ-tocopherol. Factor 2 (other nutrients) had high loadings of selenium, vitamin A (retinol), vitamin B_6_, vitamin E (α-tocopherol), lycopene, α-carotene, and β-cryptoxanthin. Factor 3 (inflammation) had high loadings of IFN-γ, TNF-α, IL-6 and IP-10 (Additional file 5: Table S5).

Interestingly, higher scores of factor 1 (high carotenoids and low IL-18) were associated with reduced hazards of TB incidence in both univariable (HR 0.56; 95% CI 0.37–0.82) and multivariable analyses (aHR 0.48; 95% CI 0.26–0.87) (Table 4). Higher scores of factor 2 (other nutrients) were associated with reduced hazards of TB incidence in univariable but not in multivariable models. Unlike the results with CTF, factor 3 (inflammation) was not associated with increased hazards of incident active TB in both univariable (HR 1.15; 95% CI 0.83–1.54) and multivariable models (aHR 0.91; 95% CI 0.57–1.45) (Table 4).

**Table 4:** Association of each factor with incident active TB

	Univariable analysis HR (95% CI)	Multivariable analysis HR (95% CI)
Factor 1 (High carotenoids and low IL-18)	0.56 (0.38–0.82)	0.48 (0.26–0.87)
Factor 2 (Other nutrients)	0.65 (0.45–0.95)	0.98 (0.48–2.05)
Factor 3 (Inflammation)	1.15 (0.83–1.54)	0.91 (0.57–1.45)

The association of each factor with incident active TB was determined in univariable and multivariable Cox regression models. Baseline variables including sex, age, country, treatment arm, body mass index (BMI), CD4 count, viral load, baseline TB status, anemia, and hypoalbuminemia were adjusted for in the multivariable models. *N* = 220 (47 cases, 173 controls)

### Sensitivity analyses

Although incident active TB meets the definition for both CTF and severe outcomes, the inclusion of TB as an element of these outcomes (CTF and severe outcomes) could potentially affect the derivation of the factors and association of the factor with the outcomes. As a result, we have conducted sensitivity analyses that remove incident active TB from the CTF (*N* = 242) and severe outcome (*N* = 208) analyses. The factor profiles were similar, and the results are consistent with the original analysis for the both the CTF (aHR 1.50; 95% CI 1.15–1.98 for factor 3) and severe outcomes (aHR 1.95; 95% CI 1.35–2.82 for factor 3) analysis (data not shown).

Our case-control analysis of incident active TB included individuals with prevalent TB at baseline (pre-ART initiation). In a sensitivity analysis removing those with prevalent TB at baseline, the profile of factors remained similar, and our results were consistent (aHR 0.46; 95% CI 0.23–0.91 for factor 1) (data not shown).

## Discussion

In our study of HIV-infected individuals initiating ART, underlying factors were extracted from multiple biomarkers of baseline nutritional and immunological status, and the association of these factors with adverse HIV outcomes (CTF and incident active TB) was assessed. Higher scores of the inflammation factor were associated with increased hazards of CTF. Interestingly, higher scores of the high carotenoids and low IL-18 factor were associated with reduced hazards of incident active TB. Our results, utilizing analytical approaches that account for correlations (e.g., EFA) between multiple biomarkers, support findings from other studies on the association of inflammation and micronutrients with HIV outcomes, while suggesting that it may be valuable to focus on potential interventions that address the underlying factor rather than any one particular biomarker.

In the CTF analysis, when we assessed the relationship of each factor with outcome, only the inflammation factor was associated with increased hazards of CTF. There is increasing evidence of inflammation being associated with adverse HIV outcomes, and the high loading markers including IL-18, CRP, and sCD14 have all been individually associated with adverse HIV outcomes in studies including those from this cohort [1–6]. Although using factors, based on measurement of multiple biomarkers, to risk stratify individuals is not practical in a clinical setting when compared to single markers that are also predictive, our results can inform interventions that seek to reduce inflammation. The utility of such an approach can be seen in assessment of food insecurity, where responses from multiple questions are reduced to a single variable (although not through EFA) of food insecurity, which has shown to be associated with various adverse health outcomes in HIV [29–31] and is a target for intervention [32]. While confirming the important role of these specific cytokines, our results also suggest that a more effective approach to reduce adverse HIV outcomes might be to focus on interventions that reduce the underlying inflammation (factor) represented by various correlated markers rather than focusing on only one of the markers which might represent a more specific type of inflammation (e.g., sCD14 for monocyte activation).

Notably the extracted factors in the incident active TB analyses were distinct from those in the CTF analyses. Factor 1 comprised high carotenoids and low IL-18, and the inflammation factor had high loadings of IFN-γ, TNF-α, IL-6, and IP-10. A major reason for the profiles of the factors being different between the CTF and TB case-control analyses is that the outcomes are different (TB accounts for 31% of the cases in CTF; 100% in TB case control). The relationship between carotenoids and IL-18 is intriguing, and some studies have shown that IL-18 levels can be affected by β-carotene metabolism [33, 34].

In our association studies, only the high carotenoids and low IL-18 factors were associated with reduced hazards of incident active TB. Given the findings that over-activation of inflammation (e.g., type I IFNs) results in increased incidence of TB [35], it is biologically plausible that inflammasome activation and increase in IL-18 could also result in higher incident TB [36]. The potential protective relationship of carotenoids with incident TB warrants further investigations, but potential mechanisms include an effect on immunity (e.g., inflammation, macrophage function, mucosal immunity) and oxidative stress [37–40]. Plasma carotenoids are considered a biomarker of fruit and vegetable intake [41, 42], and prior studies have observed that higher intakes of fruits and vegetables are protective against risk of TB [43, 44]. A new study from HIV-uninfected individuals [45] also suggests that low carotenoid levels might be associated with increased risk of TB.

The inflammation factor was not associated with development of incident active TB. A closer look at the high loading biomarkers (IFN-γ, TNF-α, IL-6, IP-10) suggests that while they are also pro-inflammatory, they have a different profile from the ones that load highly in the CTF inflammation factor (sCD14, CRP, IL-18, ferritin). The high loading biomarkers in the incident active TB inflammation factor are the classical Th1 cytokines important in anti-TB immunity [35], and they have not been consistently associated with adverse outcomes in HIV.

In our prior analyses on the association of an individual marker with an outcome (CTF or TB) using this same dataset [6, 7, 46], specific markers that were independently associated (e.g., vitamin D) with the outcome do not have significant loadings on any of the dominant factors. However, it is important to note that it is possible for a specific marker to still be independently associated with an outcome despite not being a part of one of the extracted factors. Factors are chosen based on correlations between the observed variables and are independent of the outcome; thus, the individual marker might or might not strongly load into any of the extracted factors. Future studies should test whether interventions that target the individual marker (e.g., vitamin D) and/or the underlying factor (e.g., inflammation) may improve clinical outcomes. Examples of potential therapeutics that may affect the underlying factor include diet (e.g., high-carotenoid foods or supplements for TB) and probiotics (to reduce microbial translocation and immune activation), along with medications to reduce inflammation (e.g., statins and aspirin) and treat co-morbidities (e.g., cytomegalovirus and helminth infections). Future studies will need to address how these interventions will affect the factors and ultimately HIV and TB outcomes.

The strengths of our study include the assessment of exposure prior to the outcome, the assessment of multiple outcomes, and addressing potential collinearity between multiple correlated biomarkers by using EFA. A limitation of this study is the CTF definition for the primary outcome, which is a composite definition based on multiple outcomes ranging from death to less severe outcomes. While this is based on a WHO definition that is widely used, we conducted a secondary analysis with more severe outcomes as well as virologic failure and observed a similar pattern. In this study, we are also unable to distinguish between TB recently acquired through transmission, TB reactivation, and subclinical/unmasked TB. The parent study was not designed to distinguish between TB reactivation and TB recently acquired through transmission. About 30% of the incident active TB cases developed within 3 months of ART initiation, which suggests that they might be subclinical or unmasked TB. However, we found a similar strength of associations but were underpowered to reach statistical significance at the *p* < 0.05 level when focusing our analyses only on new TB cases occurring after 3 months post-ART initiation. Another limitation of our study is that we did not assess food insecurity. Given the literature on the association of food insecurity with adverse HIV outcomes [29–31] along with its link to nutrition and immunity [47], a better understanding of the relationship between the extracted factors and food insecurity may have provided further insight into potential interventions.

## Conclusion

In conclusion, our results suggest that groupings of nutritional and immunological biomarkers underlying specific factors are associated with adverse events and that an approach focusing on interventions targeting the underlying factor rather than any single observed variable warrants investigation. In addition, our results focus on a group of inflammatory biomarkers that further confirm the central role of inflammation in adverse HIV outcomes, while also suggesting that carotenoids potentially protect against TB.

## Additional files

Additional file 1: **Table S1.** Biomarker loadings for each factor in primary CTF analysis (*N* = 290). (DOCX 18 kb)

Additional file 2: **Table S2.** Biomarker loadings for factors in severe outcomes analysis (*N* = 254). (DOCX 15 kb)

Additional file 3: **Table S3.** Biomarker loadings for factors in virologic failure analysis (*N* = 260). (DOCX 15 kb)

Additional file 4: **Table S4.** Association of each factor with virologic failure. (DOCX 14 kb)

Additional file 5: **Table S5.** Biomarker loadings for factors in incident active TB analysis (*N* = 220). (DOCX 15 kb)

## Abbreviations

ACTG: AIDS Clinical Trials Group
aHR: Adjusted hazard ratio
ART: Antiretroviral therapy
BMI: Body mass index
CI: Confidence interval
CRP: C-reactive protein
CTF: Clinical treatment failure
EFA: Exploratory factor analysis
ELISA: Enzyme-linked immunosorbent assay
HIV: Human immunodeficiency virus
sCD14: Soluble CD14
TB: Tuberculosis
