# Supplementary material for: Inflammation and micronutrient biomarkers predict clinical HIV treatment failure and incident active TB in HIV-infected adults: a case-control study
Source: BMC Med. 2018 Sep 24;16:161. doi: 10.1186/s12916-018-1150-3 (PMC6151930; doi:10.1186/s12916-018-1150-3)
Supplement: Supplementary file 1 — Table S1. Biomarker loadings for each factor in primary CTF analysis (N = 290). (DOCX 18 kb) [file 12916_2018_1150_MOESM1_ESM.docx]

**Additional file 1: Table S1** Biomarker loadings for each factor in primary CTF analysis (N=290)

| **Biomarkers** | **Factor 1**  **“Carotenoids”** | **Factor 2**  **“Other Nutrients”** | **Factor 3**  **“Inflammation”** |
| --- | --- | --- | --- |
| IFNγ |  |  |  |
| IL6 |  |  |  |
| IP10 |  |  |  |
| IL18 |  |  | 0.49 |
| TNFα |  |  |  |
| CRP |  |  | 0.54 |
| sCD14 |  |  | 0.35 |
| IgM |  |  |  |
| Ferritin |  |  | 0.59 |
| α-carotene | 0.41 | 0.39 |  |
| α-tocopherol (Vitamin E) |  | 0.52 |  |
| Vitamin B12 |  |  |  |
| Vitamin B6 |  | 0.32 |  |
| β-carotene | 0.63 |  |  |
| β-cryptoxanthin | 0.38 | 0.38 |  |
| γ-tocopherol |  |  |  |
| Lutein | 0.71 |  |  |
| Lycopene |  | 0.52 |  |
| Retinol (Vitamin A) |  |  |  |
| Selenium |  | 0.66 |  |
| Soluble Transferrin receptor |  |  |  |
| Vitamin D |  |  |  |
| Zeaxanthin | 0.79 |  |  |

Significant factor loadings (>0.30) for the biomarkers in the primary analysis (CTF analyses) are shown for the extracted factors after Varimax rotation. Three factors were extracted and named “Carotenoids”, “Other nutrients” and “Inflammation” based on the characteristics of the biomarkers with the high loadings. N=290 for individuals who had measures for all 23 biomarkers.
