# Supplementary material for: Inflammation and micronutrient biomarkers predict clinical HIV treatment failure and incident active TB in HIV-infected adults: a case-control study
Source: BMC Med. 2018 Sep 24;16:161. doi: 10.1186/s12916-018-1150-3 (PMC6151930; doi:10.1186/s12916-018-1150-3)
Supplement: Supplementary file 4 — Table S4. Association of each factor with virologic failure. (DOCX 14 kb) [file 12916_2018_1150_MOESM4_ESM.docx]

**Additional file 4: Table S4** Association of each factor with virologic failure

|  | **Univariable Analysis**  **HR (95% CI)** | **Multivariable analysis**  **HR (95% CI)** |
| --- | --- | --- |
| Factor 1 (“Carotenoids”) | 0.72 (0.55-0.95) | 0.84 (0.60-1.17) |
| Factor 2 (“Other Nutrients”) | 0.79 (0.62-1.01) | 0.77 (0.54-1.10) |
| Factor 3 (“Inflammation”) | 1.32 (1.11-1.56) | **1.36 (1.05-1.75)** |

The association of each factor with virologic failure was determined in univariable and multivariable cox regression models. Sex, age, country, treatment arm, body mass index (BMI), TB status, CD4 count, viral load, anemia and hypoalbuminemia were adjusted for in the multivariable models. Virologic failure were defined as HIV-1 RNA levels ≥1000 copies/mL for two successive visits at ≥16 weeks after ART initiation. N=260 (90 cases, 170 controls).
