# Supplementary material for: Inflammation and micronutrient biomarkers predict clinical HIV treatment failure and incident active TB in HIV-infected adults: a case-control study
Source: BMC Med. 2018 Sep 24;16:161. doi: 10.1186/s12916-018-1150-3 (PMC6151930; doi:10.1186/s12916-018-1150-3)
Supplement: Supplementary file 5 — Table S5. Biomarker loadings for factors in incident active TB analysis (N = 220). (DOCX 15 kb) [file 12916_2018_1150_MOESM5_ESM.docx]

**Additional file 5: Table S5** Biomarker loadings for each factor in secondary analysis of incident active TB (N=220)

| **Biomarkers** | **Factor 1**  **“High Carotenoids and Low IL18”** | **Factor 2**  **“Other Nutrients”** | **Factor 3**  **“Inflammation”** |
| --- | --- | --- | --- |
| IFNγ |  |  | 0.59 |
| IL6 |  |  | 0.51 |
| IP10 |  |  | 0.34 |
| IL18 | -0.35 |  |  |
| TNFα |  |  | 0.63 |
| CRP |  |  |  |
| sCD14 |  |  |  |
| IgM |  |  |  |
| Ferritin | -0.38 |  |  |
| α-carotene | 0.42 | 0.38 |  |
| α-tocopherol (Vitamin E) |  | 0.54 |  |
| Vitamin B12 |  |  |  |
| Vitamin B6 |  | 0.34 |  |
| β-carotene | 0.55 |  |  |
| β-cryptoxanthin | 0.38 | 0.43 |  |
| γ-tocopherol | -0.33 |  |  |
| Lutein | 0.64 |  |  |
| Lycopene |  | 0.51 |  |
| Retinol (Vitamin A) |  | 0.35 |  |
| Selenium |  | 0.63 |  |
| Soluble Transferrin receptor |  |  |  |
| Vitamin D |  |  |  |
| Zeaxanthin | 0.76 |  |  |

Significant factor loadings (>0.30) for the biomarkers in the secondary analysis (Incident active TB analyses) are shown for the extracted factors after Varimax rotation. Three factors were extracted and named “High Carotenoids and Low IL18”, “Other nutrients” and “Inflammation” based on the characteristics of the biomarkers with the high loadings. N=220 for individuals who had measures for all 23 biomarkers.
